# Children’s evaluations of deviant peers in the context of science and technology: The role of gender group norms and status

**DOI:** 10.1016/j.jecp.2020.104845

**Published:** 2020-07

**Authors:** Luke McGuire, Emma Jefferys, Adam Rutland

**Affiliations:** a Department of Psychology, University of Exeter, Exeter EX4 4QG, UK; b UCL Institute of Education, Bloomsbury, London WC1H 0AL, UK

**Keywords:** Computer science, Peer group norms, Intragroup dynamics, Peer evaluation, Gender stereotypes, STEM

## Abstract

Women are drastically underrepresented within computer science, which is in part informed by societal ideas of who can and should belong in the sciences. Less is known about how children evaluate their peers who challenge gendered expectations of who can and should take part in computer science. The current study asked children (*N* = 213; 110 girls) in middle childhood (*M*_age_ = 8.71 years; *n* = 108) and late childhood (*M*_age_ = 10.56 years; *n* = 105) to evaluate a gender-matched peer who challenged a group norm related to either computer science (male-gendered domain) or biology (less male-gendered domain). Male participants most negatively evaluated a peer who wanted to take part in a biology activity when the rest of the group wanted to do a programming activity. Furthermore, male participants expected their group to negatively evaluate this deviant peer in the programming condition. Mediation analysis revealed that for boys in the computer science condition, perceived group evaluation predicted individual evaluation. Female participants, in contrast, did not negatively evaluate someone who challenged a STEM (science, technology, engineering, and math) peer group norm. This study demonstrates that male peer groups may perpetuate the idea that computer science is for men through negative evaluation of in-group members who challenge those ideas and, in turn, maintain their dominant position as the high-status group. Achieving equity in the computer science field will require a greater understanding of these peer group norms.

## Highlights

• Boys negatively evaluate peers who challenge group norms related to computing.
• Children expect groups to negatively evaluate challenges to science gender norms.
• Perceptions of group evaluation predict how boys individually evaluate their peers.

## Introduction

Women are underrepresented among computer science graduates (15%) and in the computer science workforce (16%) within the United Kingdom (Women into Science and Engineering [WISE Campaign], 2018). This is indicative of the “leaky pipeline” phenomenon (Alper, 1993), where female students become increasingly less well represented at each stage of the education system and into employment. Ideas and expectations about who can and should take part in specific domains of science, technology, engineering, and mathematics (STEM) are often based on gender and develop from early childhood (Cvencek et al., 2011, Mulvey and Irvin, 2018). This can mean that girls, relative to boys, become increasingly excluded from STEM activities in school and outside of school, especially in the field of computing.

Developmental research (Killen and Rutland, 2011, Rutland et al., 2015) has shown a shift from middle childhood to late childhood, with biases not explicitly expressed but rather shown through the exclusion of peers who deviate from the norm of the group (henceforth “deviants”). This developmental trend is explained by an increasing understanding of group dynamics across this age range. The current study examined, for the first time, the development of children’s own evaluations of peers who deviate from peer group norms regarding STEM activities (e.g., boys who do not want to do computing).

The current study should inform our understanding of how such processes can contribute to the emergence of the leaky pipeline phenomenon from late childhood to early adolescence. Individuals who challenge gender expectations regarding STEM are of particular importance because they may help to establish norms that counter the leaky pipeline phenomenon. If these deviants who challenge group norms face more negative evaluation from their same-gender peers, this might help to explain why individuals choose not to pursue counterstereotypical STEM career pathways. This study aimed to explore how children in middle and late childhood evaluate their peers who deviate from a gender group norm related to STEM activities (i.e., doing computer programming or biology) and how they believe their gender in-group peers would evaluate these individuals.

### Gender peer groups and STEM

Peer groups are important in fostering STEM motivation and engagement in childhood. For example, preschool children (4–5 years) report greater self-efficacy and interest when engaging with a STEM task when placed in a meaningful peer group than when completing the same task alone (Master, Cheryan, & Meltzoff, 2017). However, gender peer group membership also comes with beliefs about ability in STEM that can be harmful to motivation and engagement. There is evidence that these beliefs about gender differences in STEM ability begin to appear in middle childhood. Between approximately 6 and 10 years of age, children endorse the stereotype that boys are better at math than girls (Cvencek et al., 2011). This cannot be explained by any measurable gender difference in ability (Lindberg et al., 2010, O’Dea et al., 2018) and may in part be attributed to societal expectations regarding STEM ability in relation to gender (Mascret & Cury, 2015).

In the United Kingdom, children now begin to learn about computer science and programming from as young as 5 years, using knowledge of algorithms and programming language to create simple scripts and programs (Computing at School [CAS], 2017). Therefore, for children between middle and late childhood, this is a highly relevant context where gendered ideas about ability or belonging may come to influence who takes part in particular STEM activities.

Furthermore, computer science remains a key area in which women are underrepresented within the United Kingdom. Even though all children are introduced to computer science at an early age in the United Kingdom, in 2019 girls aged 16 years represented only 21% of students who chose to take national exams in computer science. Moreover, in 2019 only 13% of girls aged 18 years chose to take a national exam in computer science (Women into Science and Engineering, 2019a, Women into Science and Engineering, 2019b). Taken together, these statistics suggest a leak of girls from the computer science pipeline before 16 and 18 years of age. Therefore, it is crucial to examine ideas about computer science and gender much earlier than this age range, with a particular focus on how peers who challenge gendered expectations are evaluated by their peers.

In the current study, we compared deviance in the context of a computer science activity with deviance surrounding a biology activity. Comparing similar U.K. statistics with those outlined above for computer science, in 2019 girls aged 16 years represented 50% of students who took national exams in biology and girls aged 18 years represented 50% of students who chose to take national exams in biology (Women into Science and Engineering, 2019a, Women into Science and Engineering, 2019b). By the graduate level, there is evidence that women hold more than 60% of biology majors (England & Li, 2006). Given the more equal gender representation around biology, we chose biology as a STEM activity to compare with computer science because the latter is a STEM activity with much greater gender inequality.

The statistics outlined above suggest that participation in computer science activities should be a central focus for research examining how children evaluate deviant peers in the area of STEM. One study carried out by Master, Cheryan, Moscatelli, and Meltzoff (2017) measured stereotypes related to robotics and computer programming ability among 6-year-olds. Participants endorsed the view that boys were better at robotics and programming than girls. The existence of such stereotypes by 6 years of age further suggests that this is an important STEM activity and age range in which to study intragroup deviance. These beliefs about robotics and programming were, in turn, related to lower self-efficacy and interest in computing for girls. A vital next step is to examine when these stereotypes surrounding computer science begin to inform children’s evaluations of their peers along with their perceptions of how their group might evaluate deviance within the STEM context.

### Peer evaluation within groups

Peer evaluation of deviants in the STEM context offers a potential developmental explanation for why individuals may leak from the STEM pipeline between late childhood and adolescence. Negative peer evaluation and social exclusion have been shown to have far-reaching consequences in terms of sociocognitive development (Leary, 1990). Between middle and late childhood, individuals come to understand that they belong to different groups and begin to navigate the complex group dynamics that come with this membership. Evaluating in-group peers is crucial in order to make decisions about social inclusion and exclusion (Killen, Mulvey, & Hitti, 2012). Such decisions can be made based on conventions (i.e., what is usually done by group members). Children will reject exclusion as unfair when it is based on gender or ethnicity alone (Killen, 2007). However, when the context involves ostensibly gendered activities (e.g., a ballet club), the situation is seen as more ambiguous. When only one individual can be included in such situations, children prioritize an individual who fits with the gender normative activity (e.g., girls are *usually* good at ballet) and justify this with reference to ensuring smooth group functioning (Killen & Stangor, 2001).

Peer group norms (i.e., rules or expectations that guide how group members ought to behave in certain contexts) are one of the key group processes that children rely on to guide their decision making surrounding peer exclusion and inclusion. These norms often draw the lines of group membership because those who do not adhere to the norms might not be seen as group members. As such, peer group norms are useful tools that can be used as a guide when evaluating peers. When peer group norms are related to conventions for dress (e.g., what color T-shirt group members should wear), children do not always evaluate deviance negatively, recognizing that some decisions fall within the domain of personal choice (Hitti, Mulvey, Rutland, Abrams, & Killen, 2014). So, far less is known about whether deviations from gendered norms about STEM are treated similarly as an issue of personal choice or whether children see such deviation as a threat to gender group identity and, therefore, evaluate this negatively.

Studies examining peer evaluation reveal a further important distinction between the individual perspective (i.e., “What do I think about this peer?”) and the perceived group perspective (i.e., “What do I think my group thinks about this peer?”). Beginning in late childhood (9–11 years), when children are asked what their *group* would think, they often report that their peers would evaluate deviancy less positively than they individually would (Rutland et al., 2015). This reflects an advanced understanding of group dynamics that begins to emerge in late childhood. Children understand that whereas they may individually recognize the right to personal autonomy, the group is more likely to focus on the threat that in-group deviants represent to intragroup cohesion. This developmental trend is one of the reasons for our focus on the transition between middle childhood and later childhood in the current study. Given this distinction and a developing understanding of the importance of group dynamics between middle and late childhood (Abrams, Rutland, Pelletier, & Ferrell, 2009), we expected to observe developmental differences in evaluations of a deviant peer. Specifically, we expected that in late childhood, compared with middle childhood, individuals would expect their group to more negatively evaluate a peer who deviates from the group norm.

Given the highly gendered nature of stereotypes surrounding computer science (Master et al., 2017), we also considered the importance of gender in peer evaluation. Previous work examining evaluation of in-group deviance has examined situations where peers challenge gender group norms about toy or activity choice. For example, Mulvey, Rizzo, and Killen (2016) examined evaluations of deviancy against a gender-stereotyped toy choice (e.g., boys play with toy race cars) from both the individual and perceived group perspectives in children between 3 and 6 years of age. Their study demonstrated the emerging understanding that the individual’s desire to support challenges to gender stereotypes will not always align with the group’s desire to maintain intragroup functioning. Similarly, Mulvey and Killen (2015) demonstrated that by adolescence (13–14 years) there was an expectation that whereas the individual would support challenging a gendered activity norm (e.g., girls do ballet), the group would not support this challenge.

In the current study, we extended this existing work on deviant evaluation by asking children to evaluate a deviant peer in a computer science context where existing gender group expectations are highly salient—particularly for boys, who represent the status quo within this STEM domain. Within the age range of our sample (8–11 years), boys have been shown to hold less flexible ideas about gender roles than girls (Blakemore, 2003, Levy and Taylor, 1995). Similarly, within the domain of computer science, men and boys represent the high-status majority group. We know that membership of such high-status groups is associated with prejudice toward lower status groups among children in this age range (Nesdale et al., 2004, Nesdale et al., 2005). Given this, we expected to see the most negative individual evaluations of deviance in the domain of computer science among male participants compared with female participants.

Finally, in the current study, we examined the relation between perceived group evaluations and individual evaluations of deviance from the STEM norm by peers. Previous research has documented that perceptions of group evaluations can predict how individuals evaluate their peers (McGuire, Rizzo, Killen, & Rutland, 2019); thus, in the current study, we assessed participants’ evaluations as compared with what they expected the group to think. This again speaks to the importance of intragroup dynamics within this age range, with children coming to make individual evaluations of deviant peers based on how they expect their *peers* will make the same evaluation.

We hypothesized that individual evaluations of deviants in the STEM context would be related to perceptions of how the peer group might evaluate the deviant peer. In particular, we anticipated that this relationship would be stronger for boys compared with girls. This is because boys belong to a high-status gender group that holds a privileged position in the field of computer science, holding more than 80% of higher education qualifications and jobs in this area (Women into Science and Engineering, 2018, Women into Science and Engineering, 2019c). As with many high-status groups, groups of boys, unlike girls, typically have strongly held norms about what group members should do to ensure that there is no threat to the dominance and distinctiveness of their high-status group (Blakemore, 2003, Levy and Taylor, 1995). Given this, we expected that boys, but not girls, would perceive their gender group to be especially negative toward deviants in the context of a high-status computer science activity compared with the more neutral biology activity.

Boys should typically seek acceptance from their high-status gender group and, therefore, should pay great attention to what their group expects of them when forming their own evaluations of deviant peers. Therefore, boys’ individual evaluations of a deviant were expected to closely match their expectation that their fellow group members would negatively evaluate a deviant male in-group member in the context of computer science. For girls, in contrast, we did not expect that perceived group evaluations would influence individual evaluations of the deviant in the same way. This is because we did not anticipate that what girls expected their gender group to think about the deviant would be any more negative in the context of programming compared with biology.

### The current study

Children aged 8–12 years were inducted into simulated gender groups that held an activity norm (a STEM activity that their gender group wanted to take part in). The chosen science activities were programming (seen as “for men/boys”; Master, Cheryan, Moscatelli, et al., 2017) and biology (a subject with fewer associated gender stereotypes). Participants were asked to evaluate a deviant peer (i.e., someone who wanted to do a different activity than the rest of the group). Participants evaluated the deviant peer from both their individual perspective (“What do you think?”) and their group perspective (“What would the rest of your group think?”). We also assessed participant interest in these STEM domains in order to control for this in our analyses. The central aim of this study was to understand whether evaluations of deviants who went against gender group norms in the domains of computer science and biology differed based on participant gender and age.

### Hypotheses

#### Hypothesis 1 (H1)

From an individual perspective, we expected that boys, compared with girls, would more negatively evaluate a deviant who challenged a computer science norm (*individual evaluation hypothesis*). In particular, we expected this in late childhood, with a more advanced understanding of group dynamics and awareness that deviance against a computer science norm would threaten the male group identity.

#### Hypothesis 2 (H2)

We expected participants to report that their group would negatively evaluate the deviant. In line with H1, we expected developmental and gender differences such that boys in late childhood would expect their group to be more negative than boys in middle childhood (*perceived group evaluation hypothesis*).

#### Hypothesis 3 (H3)

Finally, we expected to observe that the relationship between the science activity norm and individual evaluations of the deviant would be mediated by perceived group evaluations of the deviant. However, this was predicted only for boys *(grouped mediation hypothesis*). Specifically, when boys perceived their group would be more negative toward a deviant in the computer programming norm condition compared with the biology norm condition, they were expected to more negatively evaluate this deviant themselves.

## Method

### Participants

Participants (*N* = 213; 110 girls) were recruited either as part of a school visit to a science center or directly from schools in the Midlands and the South East of the United Kingdom. These participants were analyzed as two age groups; middle childhood (8–9 years, *M* = 8.71; *n* = 108) and late childhood (10–12 years, *M* = 10.56; *n* = 105). Participants attended schools serving low to middle socioeconomic status populations. The sample was composed of 41% White British, 15% Pakistani British, 12% Black British, 11% Bengali British, 5% mixed race/dual heritage, and 16% other ethnic groups, including Indian British and Chinese British (1% of participants did not provide ethnic group information). Power analysis for an analysis of variance (ANOVA) with eight groups was conducted in G*Power to determine a sufficient sample size using an alpha of .05, a power of .95, and a medium effect size of *η*_p_^2^ = .025 (Faul, Erdfelder, Lang, & Buchner, 2007). Based on these assumptions, the desired sample size was 210 participants.

### Design and procedure

All measures were approved by the Goldsmiths, University of London ethics committee as part of the project “The Influence of STEM Gender Group Norms.” The study used a 2 (Age: middle childhood or late childhood) × 2 (Gender: female or male) × 2 (Group Norm: programming or biology) between-participants design. All measures were completed using paper surveys, and an experimenter was available throughout the testing procedure to answer questions. The survey took approximately 20 min to complete.

Participants who were visitors to the science center took part during a prearranged school group visit in exchange for entry to the planetarium in the center later in the day. Parental consent was collected prior to the school’s visit to the site. Only children who had parental consent and gave their own assent took part (although all children in the visiting school group were given access to the planetarium). Children who did not have parental consent continued their visit in a different part of the science center while their classmates filled out the survey. Participants completed the survey in a classroom on-site during the first part of their day. The remaining participants were recruited directly from schools and completed the survey during the school day in a classroom setting. These participants also received parental consent in advance and gave their own assent to participate.

Participants were first asked to imagine that they were part of an after-school activities club that regularly meets to take part in events and projects together. The club was represented by an illustration of four gender-matched individuals, including individuals from multiple ethnic backgrounds. Participants were asked to pick a club name, T-shirt color, and logo in order to instill feelings of in-group identification (see McGuire et al., 2017, McGuire et al., 2015, Nesdale and Dalton, 2011).

### Group norm

The group norm was established by telling participants that their group had taken part in either programming or biology projects in the past. For example, if participants were in the programming norm condition, they would read the following:

“The rest of the after-school activities club is interested in programming. Programming is when you tell a computer, or a robot, or a phone what to do. They have done similar projects in the past and are interested in doing projects like this in the future.”

In contrast, in the biology norm condition, participants read the following:

“The rest of the after-school activities club is interested in biology. Biology is when you learn about plants, animals, and how the natural world works. They have done similar projects in the past and are interested in doing projects like this in the future.”

This description was accompanied by an illustration of the whole group as well as a further image of the activity they preferred (e.g., in the above example, a picture of several girls using computers - see online supplemental materials for images).

### Deviant peer

Next, participants were provided with more information regarding the deviant in-group member. When the group norm was biology, the deviant programming peer was described as follows:

“This is [name]. [Name] is really interested in robotics and computer programming. Programming is when you tell a computer, or a robot, or a phone what to do. [Name] wants the after-school activities club to work on a robot programming project this term.”

When the group norm was programming, the deviant biology peer was described as follows:

“This is [name]. [Name] is really interested in biology and plant cells. Biology is when you learn about plants, animals, and how the natural world works. [Name] wants the after-school activities club to work on a plant cell project this term.”

Each description was accompanied by an illustration of the child in question (this child was depicted as White across all conditions and participants) as well as an image of the activity the child preferred (e.g., a picture of a small robot made of Legos).

### Measures

#### Programming interest

Participants were asked “How fun is programming?” and “How fun is learning about robots?” (both questions adapted from Master et al., 2017) (1 = *not fun at all,* 6 = *really fun*). Responses to these questions positively correlated with one another (*r* = .33, *p* < .001), and as such a mean average “programming interest” score was calculated using these two items.

#### Biology interest

Participants were asked “How fun is biology?” and “How fun is learning about cells?” (1 = *not fun at all,* 6 = *really fun*). These questions were designed to mirror those about programming in the domain of biology. Responses to these questions positively correlated with one another (*r* = .42, *p* < .001), and as such a mean average “biology interest” score was calculated using these two items.

#### Individual evaluation

Participants were asked “How much do you think you would like [deviant target name]?” (1 = *not at all,* 6 = *a lot*).

#### Group evaluation

Participants were asked “How much do you think the group would like [deviant target name]?” (1 = *not at all,* 6 = *a lot*).

### Data preparation and analytic plan

First, we assessed participants’ responses to the programming and biology interest items using a 2 (Age Group: middle childhood or late childhood) × 2 (Gender: female or male) × 2 (Group Norm: biology or programming) univariate ANOVA.

Participants’ responses to the individual and perceived group deviant peer evaluation measures were then subjected to 2 (Age Group: middle childhood or late childhood) × 2 (Gender: female or male) × 2 (Group Norm: biology or programming) univariate analyses of covariance (ANCOVAs) with biology and programming interest as covariates. Follow-up simple main-effects tests were carried out where appropriate, with Bonferroni corrections for multiple comparisons applied.

To examine the connection between individual evaluations of the deviant and perceptions of group evaluation, we tested a mediation model with two groups (male and female). This model tested whether the direct effect of STEM activity norm (biology or programming) on individual evaluations of the deviant was mediated by perceived group evaluations. To test this, we specified mediation models (Model 4) with 5000 bootstraps for male and female participants using the PROCESS macro for SPSS. Participant age, biology interest, and programming interest were included as covariates in these models.

Finally, to compare these two models, we used Mplus to calculate the chi-square fit statistic for an unconstrained version of this mediation model grouped by gender (female or male) as well as a fully constrained model where each pathway was constrained to equal zero. If the difference between these two chi-square values is significant based on a right-tailed chi-square test, this would indicate that gender moderates the mediation effect. We followed the same procedure to specifically test whether the pathway between group norm and perceived group evaluation of the deviant peer was moderated by participant gender. To do so, we compared a fully constrained version of the model with one where the pathway from norm to perceived group evaluation was allowed to vary freely.

## Results

### Programming interest

First, we observed a significant main effect of gender on programming interest, *F*(1, 197) = 7.88, *p* = .005, *η*^2^ = .04. Male participants reported greater interest in programming (*M* = 5.08, *SD* = 0.99) than female participants (*M* = 4.65, *SD* = 1.23). This was further qualified by an interaction between gender and age group, *F*(1, 197) = 4.86, *p* = .03, *η*^2^ = .02. Female participants in middle childhood (*M* = 4.86, *SD* = 1.22) reported greater interest in programming than female participants in late childhood (*M* = 4.41, *SD* = 1.22), *p* = .04, *d* = 0.37. Furthermore, in late childhood, male participants (*M* = 5.21, *SD* = 0.98) reported greater interest in programming than female participants in this age group (*M* *=* 4.41, *SD* *=* 1.22), *p* = .001, *d* = .72.

### Biology interest

Again, we observed a significant main effect of gender on biology interest, *F*(1, 195) = 4.00, *p* = .05, *η*^2^ = .02. Here, female participants (*M* = 4.18, *SD* = 1.19) reported greater interest in biology than male participants (*M* = 3.84, *SD* = 1.34).

### Individual evaluation of deviant peer

Controlling for covariates [programming interest: *F*(1, 186) = 4.16, *p* = .04, *η*^2^ = .02; biology interest: *F*(1, 186) = 8.06, *p* = .005, *η*^2^ = .04], we observed a significant main effect of STEM norm activity, *F*(1, 186) = 14.41, *p* < .001, *η*^2^ = .07. Evaluations of a deviant peer who challenged a biology activity norm were significantly more positive (*M* = 4.54, *SD* = 1.26) than evaluations of a deviant peer who challenged a programming activity norm (*M* = 3.79, *SD* = 1.71).

**Fig. 1:**
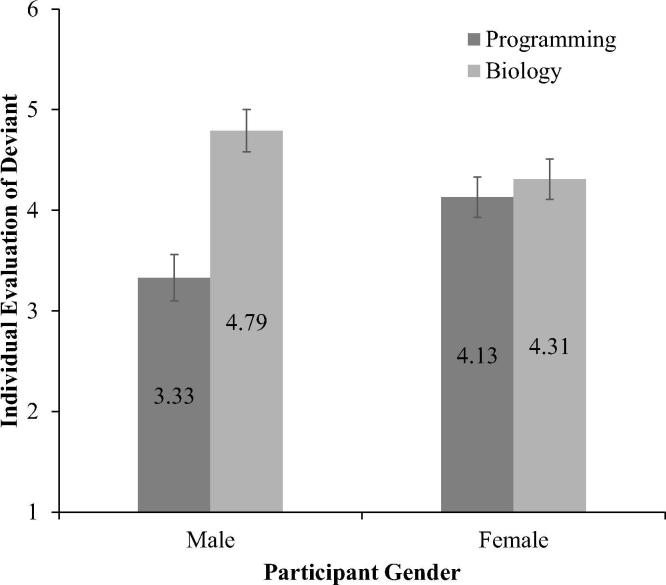
Individual evaluations of deviant target as a function of participant gender and STEM (science, technology, engineering, and math) norm activity with standard error bars (*n* = 187).

This STEM norm activity main effect was qualified by a significant interaction between gender and STEM norm activity, *F*(1, 186) = 12.09, *p* = .001, *η*^2^ = .06 (Fig. 1). For male participants, a deviant in the programming norm condition (*M* = 3.33, *SD* = 1.87) was rated less positively than a deviant in the biology norm condition (*M* = 4.79, *SD* = 1.38), *p* < .001, *d* = 0.89. In contrast, female participants’ evaluations of a deviant against a programming norm (*M* = 4.13, *SD* = 1.51) did not differ from their evaluations of a deviant in the biology norm condition (*M* = 4.31, *SD* = 1.10), *p* = .82. Furthermore, in the biology norm condition, female participants rated a deviant less positively than male participants, *p* = .05, *d* = 0.38. Inversely, in the programming norm condition, male participants rated a deviant less positively than female participants, *p* = .005, *d* = 0.47.

Thus, male participants evaluated a deviant less favorably when their group held a programming norm (i.e., a deviant who chooses biology over programming) than when their group held a biology norm (i.e. a deviant who chooses programming over biology). Female participants, in contrast, did not evaluate a deviant less favorably based on the norm condition.

### Perceived group evaluation of deviant peer

**Fig. 2:**
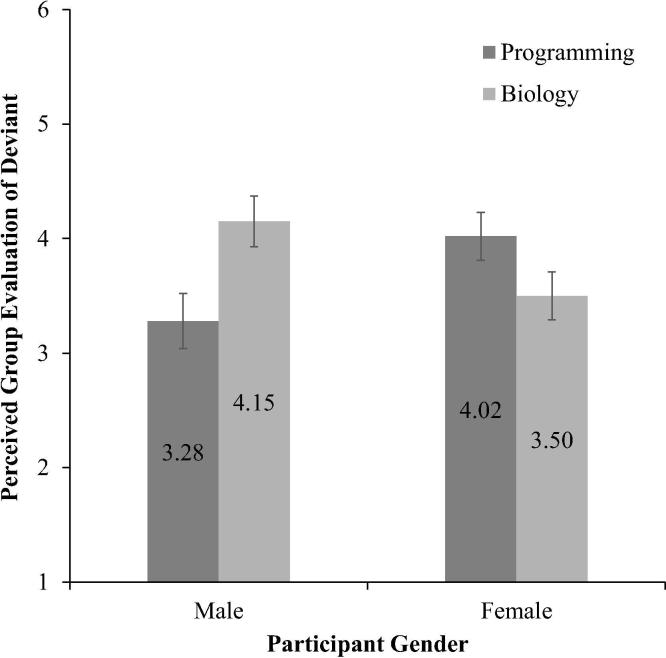
Perceived group evaluations of deviant target as a function of participant gender and STEM (science, technology, engineering, and math) norm activity with standard error bars (*n* = 180).

Controlling for covariates [programming interest: *F*(1, 179) = 0.02, *p* = .89, *η*^2^ = .00; biology interest: *F*(1, 179) = 6.94, *p* = .009, *η*^2^ = .04], when participants were asked how their group would evaluate a deviant peer, there was a significant interaction between participant gender and STEM norm activity, *F*(1, 179) = 10.74, *p* = .001, *η*^2^ = .06 (Fig. 2). Pairwise comparisons revealed that when their group held a programming norm, female participants (*M* = 4.02, *SD* = 1.53) believed their group would evaluate a deviant peer (i.e., a peer who wanted to do a biology activity) more favorably than male participants in the same condition (*M* = 3.28, *SD* = 1.76), *p* = .05, *d* = 0.35. Furthermore, male participants believed their group would evaluate a deviant in the biology norm condition (i.e. a peer who wanted to do a programming activity) more favorably (*M* = 4.15, *SD* = 1.33) than female participants in the same condition (*M* = 3.50, *SD* = 1.44), *p* = .01, *d* = 0.47. Finally, within the male participant group, participants perceived their group would evaluate deviance against a programming norm significantly less positively than deviance against a biology norm, *p* = .007, *d* = 0.56. There was no difference in evaluation between the STEM norm activity conditions among female participants, *p* = .06.

### Link between individual and group evaluations of deviant peer

#### Male participants

**Fig. 3:**
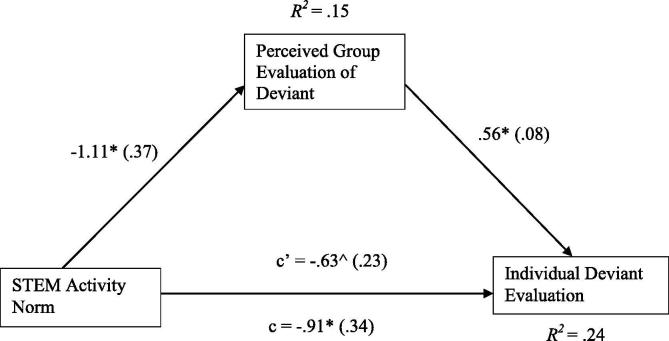
Mediation of STEM (science, technology, engineering, and math) activity norm (0 = biology, 1 = programming) and individual deviant evaluation through perceived group evaluation of deviant when gender = male (*n* = 100). Unstandardized regression coefficients are provided along the paths with error terms in parentheses. c, direct path; c’, indirect path. *p < .05. ^No zero between LLCI (lower-level confidence interval) and ULCI (upper-level confidence interval).

For male participants, the total effect model was significant, *F*(4, 78) = 6.09, *p* < .001, *R*^2^ = .24 (Fig. 3). Controlling for covariates (age: *ß* = −.14, *t* = −0.83, *p* = .41; computing interest: *ß* = .03, *t* = 0.17, *p* = .86; biology interest: *ß* = .11, *t* = 0.95, *p* = .35), the direct effect of STEM activity norm on individual deviant evaluation was significant (*ß* = −1.05, *t* = −3.26, *p* = .002, lower-level confidence interval [LLCI] = −1.69, upper-level confidence interval [ULCI] = −0.41). Coherent with the individual evaluation analysis above, when the male peer group held a programming norm, individual evaluations of a deviant were less positive.

Again controlling for covariates (age: *ß* = −.10, *t* = −0.57, *p* = .57; computing interest: *ß* = .13, *t* = 0.72, *p* = .47; biology interest: *ß* = .31, *t* = 2.47, *p* = .02), the pathway between STEM activity norm and perceived group evaluation was also significant for male participants (*ß* = −.85, *t* = −2.54, *p* = .01, LLCI = −1.52, ULCI = −0.18). When the peer group held a programming norm, perceived group evaluations of a deviant were less positive. Finally, there was a significant effect of perceived group evaluations of the deviant on individual evaluations (*ß* = .54, *t* = 5.21, *p* < .001, LLCI = 0.34, ULCI = 0.75). More positive perceived group evaluations of the deviant were related to more positive individual deviant evaluations.

Examining the indirect effect suggested that for boys the chosen STEM activity norm significantly influenced their perceived group evaluation of a deviant, which in turn predicted their individual evaluations, as indicated by the absence of a zero between the lower-level and upper-level confidence intervals (*ß* = −.46, LLCI = −0.93, ULCI = −0.06).

#### Female participants

**Fig. 4:**
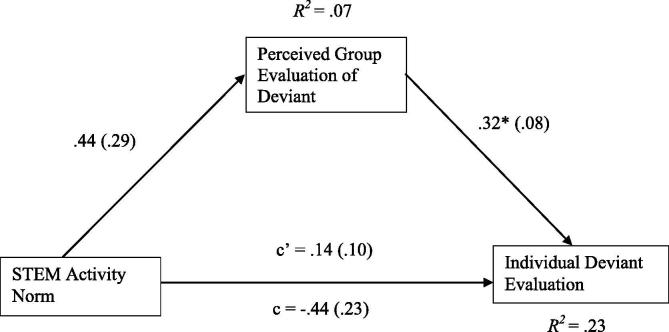
Mediation of STEM (science, technology, engineering, and math) activity norm (0 = biology, 1 = programming) and individual deviant evaluation through perceived group evaluation of deviant when gender = female (*n* = 110). Unstandardized regression coefficients are provided along the paths with error terms in parentheses. c, direct path, c’, indirect path. *p < .05.

For female participants, the total effect model was significant, *F*(4, 98) = 2.85, *p* = .03, *R*^2^ = .10 (Fig. 4). However, controlling for covariates (age: *ß* = −.003, *t* = −0.03, *p* = .98; computing interest: *ß* = .28, *t* = 2.76, *p* = .007; biology interest: *ß* = .12, *t* = 1.19, *p* = .24), the direct effect of STEM activity norm on individual deviant evaluation was not significant (*ß* = −.30, *t* = −1.25, *p* = .22, LLCI = −0.77, ULCI = 0.18).

Similarly, and again controlling for covariates (age: *ß* = −.20, *t* = −1.44, *p* = .15; computing interest: *ß* = −.03, *t* = −0.24, *p* = .81; biology interest: *ß* = .17, *t* = 1.30, *p* = .20), the pathway from STEM activity norm to perceived group deviant evaluation was not significant (*ß* = .55, *t* = 1.89, *p* = .06, LLCI = −0.03, ULCI = 1.14). The pathway between perceived group evaluation of the deviant and individual evaluation was significant (*ß* = .32, *t* = 3.97, *p* < .001, LLCI = 0.16, ULCI = 0.48). However, there was no evidence for an indirect effect of STEM norm activity on individual evaluation through perceived group evaluation (*ß* = .18, LLCI = −0.02, ULCI = 0.43).

### Model comparison

We calculated the difference between the chi-square and degrees of freedom for the constrained model, *χ*^2^(12) = 36.04, and the unconstrained models, *χ*^2^(4) = 12.92, and calculated the right-tailed probability of this chi-square distribution. This calculation suggested moderation by the grouping variable of gender in the unconstrained model, *χ*^2^(8) = 23.12, *p* < .001. To test our hypothesis that participant gender would moderate the effect of the STEM norm activity on participants’ perceived group evaluations, we used the same procedure to calculate the difference between the fully constrained version of the model, *χ*^2^(12) = 36.04, and one where the pathway from STEM activity norm to perceived group evaluation was allowed to freely vary, *χ*^2^(11) = 31.22. Again, the right-tailed probability of the chi-square distribution suggested that the pathway between STEM activity norm and perceived group evaluation was moderated by participant gender, *χ*^2^(1) = 4.82, *p* < .03.

Taken together, these models demonstrate that the influence of STEM activity norm on individual evaluations was in part mediated by perceived group evaluations for male participants. When the peer group held a programming norm, male participants perceived their group would evaluate a deviant less positively, which in turn predicted a more negative individual evaluation.

## Discussion

The findings of the current study indicate that in the domain of computer science, gender group norms inform not only how boys evaluate their peers but also how they expect their peers will evaluate others who challenge such norms. This study shows, for the first time, that STEM domain and participant gender effect the link between the individual and perceived group evaluations of deviant peers. In the context of computer science (not biology), it is only for boys (the high-status group) that more negative individual evaluations of a deviant peer were influenced by perceptions that the rest of the group would negatively evaluate this individual. First, these findings emphasize that in the realm of computer science, boys negatively evaluate their peers who challenge the boundaries of a stereotyped group activity. Second, for boys in this STEM context, peer evaluations reflect not only their understanding of broader societal expectations but also the importance of maintaining intragroup functioning. This has important consequences for extending our understanding of the leaky pipeline phenomenon. Through negative evaluation (and potential social exclusion) of those who challenge established gender norms, boys can ensure that they maintain greater numerical representation within this domain, sustain stereotypes about male ability, and reduce challenges to the status quo from within their gender in-group.

These findings extend previous work that has documented the gendered nature of computer science (Master et al., 2017) by demonstrating that boys not only see a computer programming activity as being for their own gender group but also will harshly evaluate someone who wishes to engage with *other* STEM domains when the group holds a norm for programming. The degree of negativity shown by boys in the current study is worthy of note. In comparable work examining peer evaluation, individual evaluations were generally more positive than perceived group evaluations (McGuire et al., 2019, Mulvey and Killen, 2015, Mulvey et al., 2016, Rutland et al., 2015). In contrast, individual evaluations of a deviant in the programming condition among boys did not differ from their perceived group evaluations. For boys, ensuring group functioning through adherence to STEM gender norms is of particular importance. In the context of computer science where stereotypes suggest that boys have an innate ability (Master et al., 2017), the presence of a deviant is likely to seem particularly threatening to ongoing group functioning.

When evaluating the deviant peer from both the individual and perceived group perspectives, girls did not differentiate their evaluations based on the group norm. However, girls did report greater interest in biology than their male peers and evaluated a deviant who wanted to take part in a biology activity more positively than boys. This suggests that girls may see biology and life sciences as more “for them” and, therefore, will presume their group will not negatively evaluate a peer who wishes to engage with this activity even if this threatens intragroup functioning. Alternatively, girls may see computer science as a uniquely male domain and, therefore, show less concern about deviance against a programming activity norm. Future research should aim to tease apart this distinction in order to understand which activities girls see as being for them and, in turn, how these perceptions can help to explain the leaky pipeline phenomenon, particularly within the domain of computer science. Individually, girls did not negatively evaluate a deviant peer who wanted to take part in a programming activity. Instead, it appears the normative boundaries for computer science are enforced at the group level by an expectation that female peers will less positively evaluate those who wish to pursue programming when the rest of the group acts in accordance with societal expectations for girls (i.e., want to do biology).

In the current study, we did not observe differences in individual or perceived group evaluation as a function of age. Although we expected that boys in late childhood in particular would negatively evaluate a deviant who challenged a programming norm, our results suggest that this negative evaluation is present in middle childhood (8–9 years). Previous work examining challenges to gender stereotypes has demonstrated that once they develop theory-of-mind abilities, children younger than those in our sample are able to understand that their gender peer group may negatively evaluate deviance (Mulvey et al., 2016). This study extends this field of research by demonstrating that in the context of STEM, where gender inequalities are present from a young age, members of different gender groups in middle childhood will expect their groups to evaluate a deviant more or less positively depending on the individual's relative status. This has important consequences for when educational interventions around STEM should be targeted. By 8 years of age, boys already expect their group to enforce intragroup adherence to stereotypical norms. Therefore, messages that challenge these ideas are likely to be most effective prior to this age range.

### Limitations and future directions

This study presents a number of interesting future directions for research that will also overcome some of the limitations of the methodology. In the current study, participants were inducted into same-gender STEM activity groups and asked only to evaluate a deviant peer from their own gender group. Previous work has examined how out-group deviants are evaluated from the perspective of the in-group. This can lead to the “black sheep effect” (see Marques & Paez, 1994), where an out-group deviant who agrees with the in-group norm is positively evaluated. This is a developmental effect that is known to emerge across the age range sampled here (Abrams, Palmer, Rutland, Cameron, & Van de Vyver, 2014). This phenomenon is of particular importance in the context of STEM, specifically with regard to how girls who wish to take part in computer science activities are evaluated by boys. It is currently unknown whether these girls would be positively evaluated by boys due to their shared interest (i.e., the black sheep effect) or whether boys will negatively evaluate girls who wish to take part in a STEM activity that is seen as “for boys.” Therefore, future work is required with a fully crossed design allowing us to understand how boys and girls evaluate in-group *and* out-group deviants in gendered STEM contexts. Understanding how girls who pursue such activities are evaluated by boys will be an important task for those interested in promoting equity in STEM education and employment through reducing the leak in the pipeline.

In the current study, the illustrations of the peer group and the deviant peer were not matched to the ethnicity of the participant. Whereas the peer group featured illustrations of individuals from multiple ethnic backgrounds, the deviant peer was always depicted as White. Although we did not make predictions based on the ethnicity of the deviant or the participant, there is important evidence that recognizes the intersectional nature of ethnicity and gender in relation to STEM (Charleston et al., 2014, O’Brien et al., 2015). Future work that manipulates the ethnicity of the peer group members and deviant peer is necessary in order to examine how this interacts with the ethnicity and gender of the participant. Furthermore, understanding how issues such as socioeconomic status and science capital (Archer et al., 2014, Dawson, 2014) interact with these identities will be crucial to develop the richest picture of who is included in different STEM settings. This will afford an important understanding of how multifaceted identities interact to predict intragroup understanding within STEM domains.

Examining the transition from late childhood to early adolescence will be an important next step for work in this area. Adolescents begin to make important decisions about course enrollment that inform their future career trajectories. If similar or related group processes are at work in adolescence, as evidenced in the current study, it is possible that adolescent girls may feel they risk negative evaluation by their peers if they pursue STEM pathways that are seen as traditionally male. Furthermore, understanding how and when these group norms affect STEM engagement and motivation will be a key next step. For instance, it will be important to understand whether negative evaluation from peers directly informs interest and self-efficacy in computer science. Finally, the current work did not include potential moderating variables of individual and perceived group evaluations. For example, it would be interesting for future work to examine whether in-group identification (how strongly participants identify with their gender group) influences this process. Specifically, measuring identification as a moderator of this effect will help us to understand whether the observed negative evaluations of in-group deviants are driven by girls and boys who more strongly identify with their gender in-groups.

### Conclusion

The findings of the current study extend our knowledge of peer group processes in the context of STEM and gender in a number of interesting directions. First, this study establishes that girls do not individually negatively evaluate deviancy against a gender group STEM norm. However, both boys and girls understand that their peers will negatively evaluate a deviant who challenges a group norm that is seen as gendered at the societal level (e.g., programming for boys). Furthermore, this study demonstrates that the importance of gender norms in STEM is amplified for boys, who are a high-status group within this area. Given this high status, when boys seek to pursue activities outside of those STEM areas seen as traditionally male, they may face negative evaluation. Gender group norms and group status have clear consequences for the evaluation of individuals who seek to take part in activities counter to normative expectations. Future work will be essential to challenge these norms, especially in high-status groups, at both the peer and societal levels in order to ensure equitable access to STEM educational opportunities from late childhood onward.

## CRediT authorship contribution statement

**Luke McGuire:** Conceptualization, Methodology, Formal analysis, Investigation, Writing - original draft, Writing - review & editing. **Emma Jefferys:** Methodology, Investigation, Writing - review & editing. **Adam Rutland:** Conceptualization, Methodology, Writing - review & editing.

## Funding

This work was supported collaboratively by the Wellcome Trust [grant number: 206259/Z/17/Z] and the Economic and Social Research Council.
