# Supplementary material for: Children’s evaluations of deviant peers in the context of science and technology: The role of gender group norms and status
Source: J Exp Child Psychol. 2020 Jul;195:104845. doi: 10.1016/j.jecp.2020.104845 (PMC7193893; doi:10.1016/j.jecp.2020.104845)

**Pictures accompanying peer who wants to do programming activity**


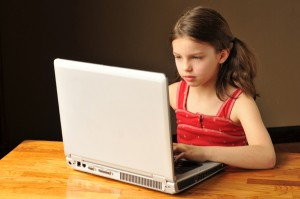

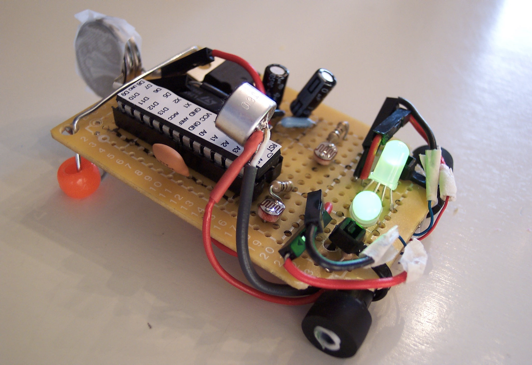


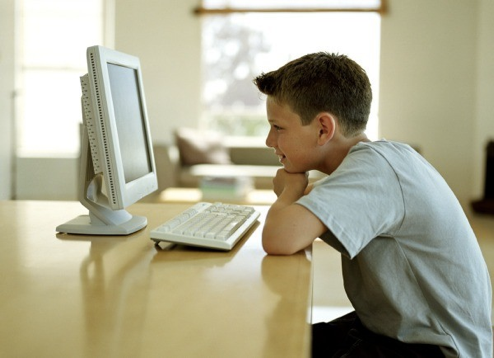


**Pictures accompanying peer who wants to do biology activity**


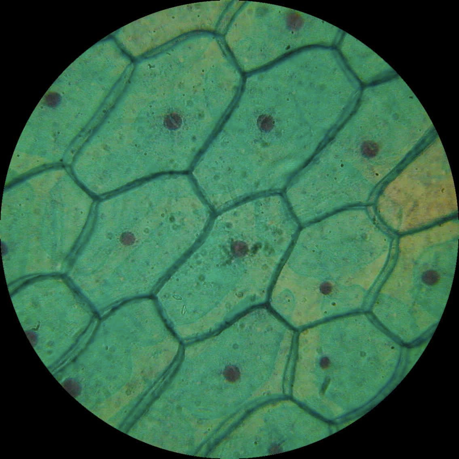

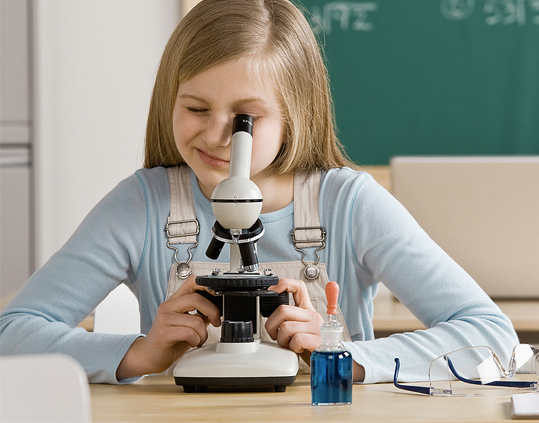


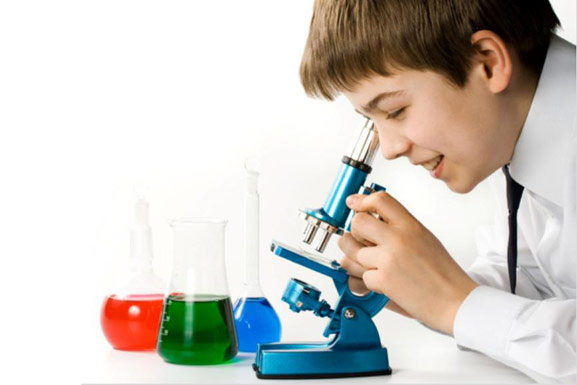


**Peer group programming image**


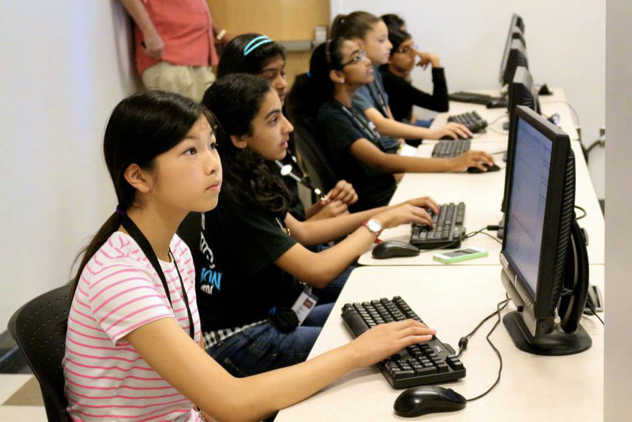

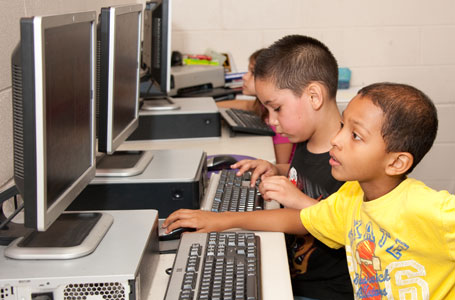


**Peer group biology image**


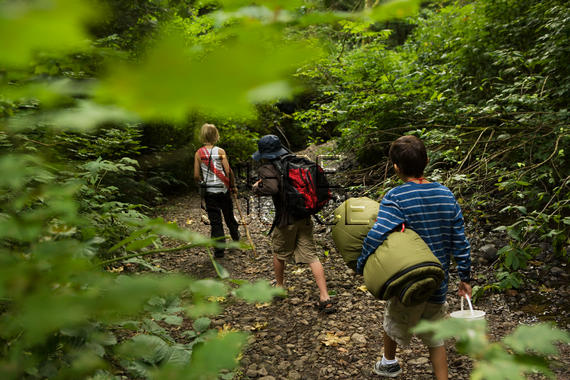

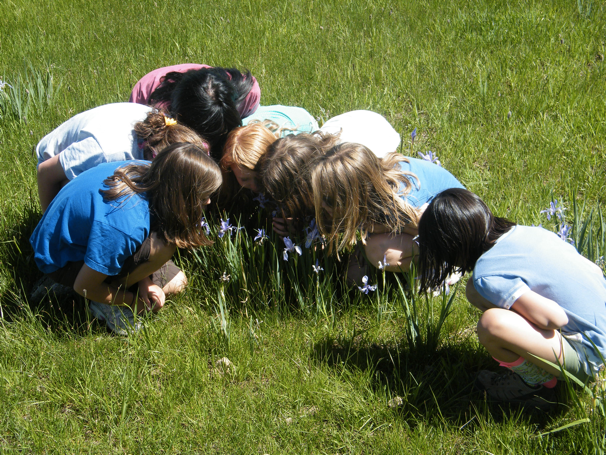

Supplement: Supplementary data 2 [file mmc2.docx]
